# Illuminating the Connection: Cutaneous Vitamin D_3_ Synthesis and Its Role in Skin Cancer Prevention

**DOI:** 10.3390/nu17030386

**Published:** 2025-01-22

**Authors:** Nazlı Uçar, Michael F. Holick

**Affiliations:** 1 Section of Endocrinology, Diabetes, Nutrition and Weight Management, Department of Medicine, Boston University Chobanian & Avedisian School of Medicine, Boston, MA 02118, USA; nucar@bu.edu; 2 Area of Preventive Medicine and Public Health, Department of Preventive Medicine and Public Health, Food Sciences, Toxicology, and Legal Medicine, School of Pharmacy, University de Valencia, 46100 Burjassot, Spain

**Keywords:** vitamin D, 25-hydroxyvitamin D, 1,25-dihydroxyvitamin D, photoprotection, non-melanoma skin cancer, ultraviolet B radiation, sunlight, DNA damage, DNA repair

## Abstract

Sunlight exposure plays an important role in human health, impacting processes such as mood, blood pressure regulation, and vitamin D_3_ production. Solar ultraviolet B radiation initiates vitamin D_3_ synthesis in the skin, which is subsequently metabolized into its biologically active form. UVB exposure plays a key role in enabling vitamin D_3_ synthesis, but it can also contribute to skin carcinogenesis, creating a complex interplay between its beneficial and harmful effects. Vitamin D deficiency, affecting over half the global population, is linked to a range of chronic diseases, including cancers, cardiovascular conditions, and autoimmune disorders. Simultaneously, excessive solar UVB exposure increases the risk of non-melanoma and melanoma skin cancers through mechanisms involving DNA damage and oxidative stress. This review examines the dual role of UVB radiation in health and disease, focusing on the mechanisms of cutaneous vitamin D_3_ synthesis, the epidemiology of skin cancer, and the protective roles of vitamin D_3_’s photoproducts and its active metabolite, 1,25-dihydroxyvitamin D_3_. Understanding these interconnections is critical for developing strategies that balance adequate sun-induced vitamin D_3_ production with skin cancer prevention.

## 1. Introduction

**Figure 1:**
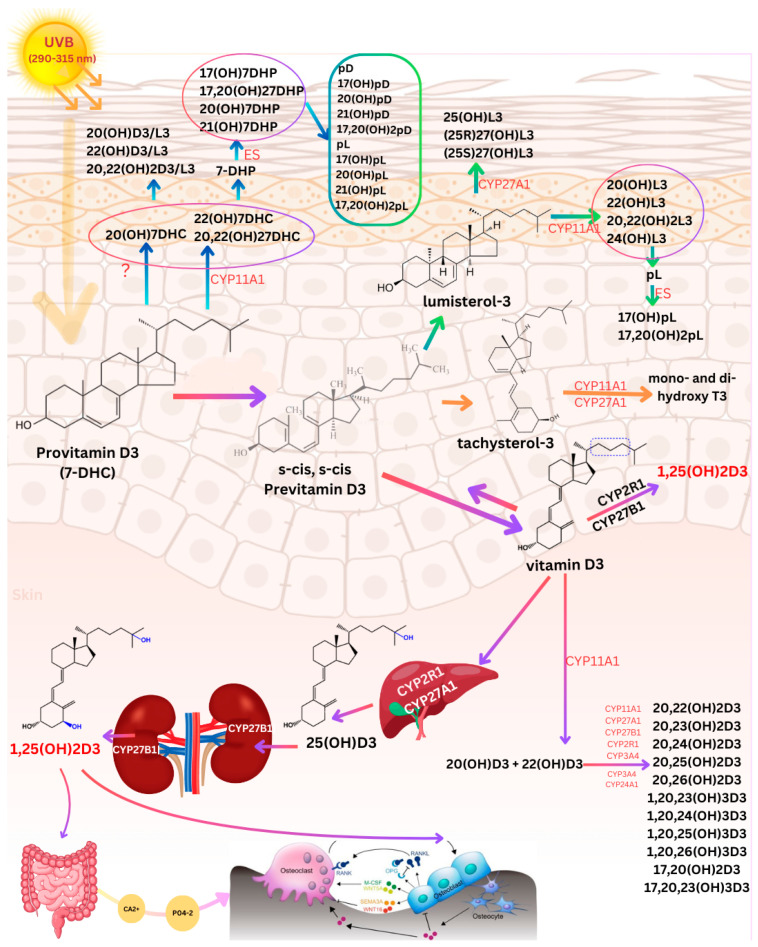
Pathway of Vitamin D_3_ synthesis and metabolism in the skin, liver, and kidneys along with non-canonical pathways of vitamin D_3_, lumisterol_3_, and tachysterol_3_ activation. Exposure to solar ultraviolet B radiation (UVB; 290–315 nm) initiates the conversion of 7-dehydrocholesterol (7-DHC) in the skin to previtamin D_3_, which is then converted to vitamin D_3_ in a heat-dependent process. Excessive solar UVB exposure can degrade both previtamin D_3_ and vitamin D_3_ into inactive photoproducts. Vitamin D_3_, lumisterol_3_ (L_3_), tachysterol_3_ (T_3_), and 7-DHC act as substrates for the enzyme CYP11A1, which, either independently or in cooperation with other cytochrome P450 (CYP) enzymes, produces the corresponding hydroxylated derivatives. In the case of L_3_ and 7-DHC, CYP11A1 can cleave the side chain to produce 7-dehydropregnenolone (7-DHP) or pregnalumisterol (pL), which are further metabolized by steroidogenic enzymes (ES). In the skin, UVB radiation acting on 5,7-dienes leads to the production of D_3_, L_3_, and T_3_ derivatives with a full-length side chain, as well as pregnacalciferol (pD), pL and pregnatachysterol (pT) derivatives with a shortened side chain. Vitamin D_3_ is then transported into the bloodstream, where it binds to the vitamin D binding protein (DBP), which facilitates its delivery to the liver. In the liver, vitamin D_3_ is hydroxylated by either CYP2R1 or CYP27A1 to form 25-hydroxyvitamin D_3_ [25(OH)D_3_], an inactive precursor. This form must undergo further hydroxylation in the kidneys by CYP27B1 to produce the biologically active form, 1,25-dihydroxyvitamin D_3_ [1,25(OH)_2_D_3_]. The active metabolite regulates calcium and phosphate homeostasis by enhancing intestinal absorption. In osteoblasts, 1,25(OH)_2_D_3_ binds to its receptor, promoting the expression of the receptor activator of nuclear factor-κB ligand (RANKL). RANKL then binds to its receptor, RANK, on preosteoclasts, triggering their differentiation into mature osteoclasts. Mature osteoclasts remove calcium and phosphorus from the bone, helping maintain blood concentrations of calcium (Ca^2+^) and phosphorus in the blood, which are critical not only for skeletal mineralization but also for most metabolic functions. Adequate Ca^2+^ and phosphorus (HPO_4_^2−^) levels promote the mineralization of the skeleton. Abbreviations: 7-DHC, 7-dehydrocholesterol; L_3_, lumisterol; T_3_, tachysterol; CYP, cytochrome P450 enzyme; 7-DHP, 7-dehydropregnenolone; pL, pregnalumisterol; ES, steroidogenic enzymes; pD, pregnacalciferol; pT, 25(OH)D_3_, 25-hydroxyvitamin D_3_; 1,25(OH)_2_D_3_, 1,25-dihydroxyvitamin D_3_; RANKL, receptor activator of nuclear factor-κB ligand; Ca^2+^, calcium; PO_4_^3−^, phosphate; ?, enzyme responsible unknown. Copyright Holick 2025.

Sunlight exposure provides numerous health benefits including stimulating the expression of the pro-opiomelanocortin (POMC) gene, which produces beta endorphins, natural opioids that can elevate mood and contribute to the feeling of well-being [1,2,3]. Sunlight triggers the production and release of nitric oxide (NO), resulting in a reduction in blood pressure, which may explain why higher latitudes are associated with higher blood pressure and increased risk of cardiovascular mortality [4]. Another critically important role of sunlight is its stimulation of cutaneous vitamin D_3_ synthesis, commonly known as the “sunshine vitamin”. Vitamin D_3_, a vital prohormone for human health, is synthesized in the skin after exposure to solar ultraviolet B radiation (UVB, 290–315 nm). This process begins in the skin when solar UVB protons are absorbed by 7-dehydrocholesterol (7-DHC) to form previtamin D_3_, which is subsequently isomerized into vitamin D_3_ by body temperature. After its formation, vitamin D_3_ is translocated from the skin into the circulation where it undergoes further metabolism to become biologically active to regulate calcium, phosphorus, and bone metabolism [1] (Figure 1).

The consequences of vitamin D deficiency not only include bone disorders such as rickets, osteomalacia, and osteoporosis but also a multitude of chronic illnesses including heightened risks of various cancers, autoimmune diseases, infectious diseases, and cardiovascular conditions [5,6,7,8,9,10,11,12,13,14,15,16,17,18,19,20,21,22,23,24,25,26,27,28,29,30,31,32,33,34,35,36]. Epidemiologic and observational studies have associated sunlight exposure to a reduced risk of many of these chronic diseases. Living at lower latitudes, where UV exposure is greater, is linked to a reduced risk of developing fatal cancers such as prostate, colon, and breast cancer, as well as autoimmune diseases including multiple sclerosis, type 1 diabetes, and infectious diseases [14,15,16,17,18,19,20,21,22]. Furthermore, birth season has also been implicated in health outcomes; for example, individuals born at the end of the winter show an increased risk of developing schizophrenia later in life [23,24]. Many of these observations, supported by association studies, indicate that improvement in vitamin D status reduces risk for these many chronic illnesses [25,26,27,28,29,30,31,32,33,34], although other studies have not come to the same conclusion [37,38]. However, a recent subgroup analysis of the VITAL trial [34,39] found that participants who took 2000 IUs of vitamin D_3_ per day experienced a 22% reduction in the risk of various autoimmune diseases, including polymyalgia rheumatica, rheumatoid arthritis, autoimmune thyroid disease, inflammatory bowel disease, systemic vasculitides, and psoriasis [34,40]. In addition, the D2d study revealed that subjects receiving 4000 IUs of vitamin D_3_ per day, which maintained a serum 25(OH)D concentration of at least 50 ng/mL (compared with 20–29 ng/mL), experienced a 76% reduction in diabetes risk, with an absolute risk reduction of 18.1% over three years [34,41,42,43].

Humans have relied on sunlight as their primary source of vitamin D_3_ throughout their evolution. The efficiency of its synthesis is influenced by multiple factors, including sunscreen use, skin pigmentation, geographic latitude, time of day, season, and aging. These variables can limit UVB availability, compounding the global issue of vitamin D deficiency [1]. Currently, more than half of the global population is at risk of vitamin D deficiency or insufficiency [34,35,36]. This deficiency is partly due to insufficient dietary intake and inadequate food fortification, and misconceptions about avoiding all direct exposure to sunlight to prevent skin cancer contribute to this widespread problem [1].

While UVB radiation is essential for initiating cutaneous vitamin D_3_ synthesis, it is also a significant factor in the pathogenesis of non-melanoma skin cancers, underscoring a complex interplay between its health-promoting and potentially harmful effects. Addressing this issue requires a comprehensive approach that prioritizes safe and controlled sun exposure to ensure adequate vitamin D_3_ synthesis while mitigating the risk of UVB-induced skin damage.

Skin cancers are among the most frequently diagnosed cancers globally, with over 1.5 million new cases reported in 2020, and projections suggest a further increase by 2040 [44]. Overexposure to sunlight will increase the risk of non-melanoma skin cancers [45,46,47,48] as both UVB and UVA irradiation induce DNA damage and oxidative stress, which promote mutations that drive the carcinogenic process [49]. The deadliest form of skin cancer, melanoma, has also been associated with exposure to UVB radiation, similar to non-melanoma skin cancers [50]. While the association of sunlight exposure with melanoma is controversial, emerging evidence suggests that regular sun exposure, particularly in occupational settings, may provide health benefits, including reduced all-cause mortality, underscoring the multifaceted effects of sunlight beyond vitamin D synthesis [51]. These findings challenge the view that all UVB exposure is harmful and highlights the complexity of sunlight’s effects on health.

The goal of this review is to explore the multifaceted role of UVB radiation in generating cutaneous vitamin D_3_ and how this relates to skin cancer by addressing the following key areas: (1) the mechanisms of cutaneous synthesis and translocation of vitamin D_3_ into the circulation, (2) the epidemiology and molecular basis of skin cancer, with focus on the relationship between sun exposure and skin cancer risk, and (3) the potential protective and preventive roles of vitamin D_3_, its metabolites, and its photoproducts.

## 2. Photoproduction and Cutaneous Metabolism of Vitamin D_3_ and Photoproducts

### 2.1. Cutaneous Transformation of 7-Dehydrocholesterol to Vitamin D_3_

Ultraviolet radiation (UVR), a component of the solar emission spectrum, encompasses wavelengths from 100 nm to 400 nm. UVR is subdivided into three categories: UVA (315–400 nm), UVB (280–315 nm), and UVC (100–280 nm). Most solar UVR is absorbed by the ozone layer, with only wavelengths at or above 290 nm reaching the Earth’s surface [52]. Thus, UVB radiation that reaches the Earth’s surface is crucial for initiating the cutaneous synthesis of vitamin D_3_ (cholecalciferol) in most vertebrates, including humans. Vitamin D_2_ (ergocalciferol), is synthesized by fungi and plants exposed to UVB radiation [53,54].

During exposure to sunlight, solar UVB photons with energies between 290–315 nm penetrate the epidermis and dermis, where these energetic photons are absorbed by 7 dehydrocholesterol (7-DHC, provitamin D_3_) in the plasma membrane of skin cells [1]. This photochemical reaction excites the double bonds in 7-DHC, causing the opening of the B-ring between carbon 9 and carbon 10. This transforms the rigid steroid structure into a more flexible molecule known as previtamin D_3_. Previtamin D_3_ exists in two conformations: s-cis, s-cis and s-cis, s-trans. The s-cis, s-cis form is energetically unstable due to steric interactions with the carbon 19 methyl group, whereas the cis–trans form is thermodynamically stable and unable to convert to vitamin D_3_. Only the thermodynamically less stable s-cis form is primarily produced in the plasma membrane of skin cells, principally in the epidermis, and therefore is rapidly transformed into vitamin D_3_ [1,55,56,57,58,59] (Figure 1).

There is ongoing debate about whether dietary vitamin D_3_ is equivalent to vitamin D_3_ that is synthesized in the skin, as vitamin D_3_ produced in the skin has a longer half-life in the circulation compared to orally ingested vitamin D_3_. Orally ingested vitamin D_3_ raises circulating concentrations of vitamin D_3_ rapidly reaching a peak concentration at approximately 12 h and returning to near baseline concentrations within 72 h. Exposure to UVB radiation results in a gradual increase in circulating concentration of vitamin D_3_ reaching a peak at approximately 12 h that is sustained at the same concentration for at least 72 h and gradually declines reaching baseline levels by 7 days [1]. There are several explanations for this difference. Firstly, previtamin D_3_ made in the living cells of the epidermis requires conversion to vitamin D_3_, which takes several hours. Vitamin D_3_ exits the plasma membrane into the extracellular space, slowly migrating through the epidermis into the dermal capillary bed. The third reason is that 100% of vitamin D_3_ entering the circulation is associated with the vitamin D binding protein (DBP), whereas when vitamin D_3_ is ingested, only about 60% is associated with the DBP. The other 40% is in the lipoprotein-bound fraction and therefore is rapidly removed from the circulation [60].

### 2.2. Production and Metabolism of Photoproducts

The triene systems in previtamin D_3_ and vitamin D_3_ can absorb UVB radiation causing them to isomerize into a variety of photoproducts (Figure 1). Previtamin D_3_ is initially isomerized to lumisterol and tachysterol, which have no calcemic activity. This photochemical process effectively limits the cutaneous production of previtamin D_3_ from excessive exposure to sunlight, thereby preventing excess vitamin D_3_ from forming and causing toxicity. This helps to explain why vitamin D toxicity has not been observed from sun exposure [1]. Additional exposure to previtamin D_3_ and vitamin D_3_ to UVB radiation results in the production of several suprasterols and toxisterols. Notably, suprasterols 5 and 6 were found to be potent inhibitors of keratinocyte proliferation [1]. These results suggest that sunlight exposure produces vitamin D_3_ and multiple biologically active photoproducts in the skin [1,61,62]. Slominski et al. [62] discovered that novel metabolic pathways initiated by CYP11A1 (cytochrome P450scc) in the skin result in the production of metabolites with biologic activity. These pathways involve the conversion of 7-DHC and vitamin D_3_ into several biologically active hydroxylated derivatives, including 20,23-dihydroxyvitamin D_3_, 20-hydroxylumisterol, and 20-hydroxyvitamin D_3_ (Figure 1). These metabolites have been reported to have several biological functions, including anti-inflammatory, anti-proliferative, and anti-cancer effects, which contribute to both local and systemic regulation of vitamin D homeostasis. Importantly, these metabolites do not exhibit hypercalcemic effects, even at relatively high concentrations. This characteristic makes them particularly valuable for therapeutic applications, especially in preventing and managing hyperproliferative skin disorders and skin cancers such as melanoma [63].

### 2.3. Factors Controlling Cutaneous Vitamin D Synthesis

The skin’s ability to produce vitamin D_3_ is influenced by various endogenous and environmental factors, including sunscreen use, clothing, skin pigmentation, season, time of day, latitude, altitude, and age. Melanin, the pigment that determines skin color, evolved as a natural sunscreen. It is highly effective at absorbing both UVB and UVA radiation, thereby protecting vital macromolecules, such as DNA, RNA, and proteins, from the harmful effects of excessive UVR exposure. Therefore, melanin efficiency in absorbing UVB radiation significantly reduces the number of UVB photons absorbed by 7-DHC, resulting in decreased production of vitamin D_3_. In individuals with darker skin—such as Africans and African Americans with skin types V and VI (which never burn and always tan)—melanin is so effective at absorbing UVB that it reduces the production of previtamin D_3_ in the skin by 95 to 99% compared to Caucasians with skin type II (who always burn and sometimes tan) [1]. As a result, individuals with darker skin tones (skin types V and VI) require 5 to 10 times more sun exposure to produce similar concentrations of previtamin D_3_ compared to those with lighter skin types (e.g., type II). According to the recent comprehensive evaluation of vitamin D status in the American population, using National Health and Nutrition Examination Survey (NHANES) data (2001–2018), the prevalence of serum 25-hydroxyvitamin D [25(OH)D, which is a measure of vitamin D status, in concentrations of <10, 10–20, 20–30, and >30 ng/mL was 2.6%, 22.0%, 40.9%, and 34.5%, respectively, among Americans aged > 1 year [64]. A circulating concentration of 25(OH)D of at least 30 ng/mL is considered necessary for maximum bone health [34,35].

Sunscreen use also impedes the production of vitamin D_3_ by absorbing UVB radiation on or near the surface of the skin, thereby decreasing the amount reaching the stratum basale and stratum spinosum, the principal layers where most vitamin D_3_ synthesis occurs. A sunscreen with a sun protection factor (SPF) of 30 absorbs 97.5% of UVB rays, thereby reducing vitamin D_3_ synthesis by 97–98% when applied properly [65]. Another important factor influencing vitamin D_3_ production is the zenith angle of the sun. As the sun’s angle becomes more oblique, the atmospheric path length increases, thereby absorbing more UVB photons and reducing the number of UVB photons that reach the Earth’s surface. This effect is particularly relevant for individuals living above approximately 35° N and below 35° S latitude, where minimal or no vitamin D_3_ is synthesized during the winter months due to reduced UVB exposure [1,52,66]. This is also the explanation for why little or no significant vitamin D_3_ is generated in the skin in the summertime before 10 AM or after 3 PM, even near the equator, due to the oblique solar angles during these times [1].

Cloud cover significantly influences cutaneous vitamin D_3_ synthesis. Overcast conditions reduce UVB irradiation reaching the Earth’s surface by more than 80%, limiting the potential for vitamin D_3_ production [67]. However, higher altitudes can enhance UVB exposure since there is less ozone to absorb UVB radiation and thus vitamin D_3_ synthesis is enhanced [1,67].

Another key consideration is the natural age-dependent decline of 7-DHC in the epidermis, which directly impacts vitamin D_3_ synthesis [1]. 7-DHC concentrations in the epidermis in younger adults (ages 20–30) can be as much as three times higher than in older adults (ages 62–80), underscoring the significant reduction in the skin’s ability to produce vitamin D_3_ as a person ages [68].

### 2.4. Metabolism of Vitamin D_3_

Biologically inactive vitamin D_3_ undergoes a two-step hydroxylation process. Initially, vitamin D_3_ is bound to the DBP in the bloodstream and is transported to the liver. In the liver, it is converted to 25(OH)D_3_ (calcidiol), primarily by the cytochrome P450 enzyme, vitamin D-25-hydroxylase (CYP2R) [35,69]. This is the major circulating form of vitamin D used to determine a person’s vitamin D status [35,70]. The preferred healthful circulating concentration has been reported to be a minimum of 30 ng/mL with a suggested desirable range of 40–60 ng/mL, as recommended by the 2011 Endocrine Society guidelines [70]. Despite being the major form in circulation, it is biologically inactive and requires further activation. The second hydroxylation occurs mainly in the kidneys, where 25(OH)D_3_ is converted to 1,25-dihydroxyvitamin D_3_ [1,25(OH)_2_D_3_; calcitriol], the biologically active hormonal form of vitamin D_3_. This conversion is controlled by the cytochrome P450 enzyme, 25-hydroxyvitamin D-1α- hydroxylase (CYP27B1) [35,36] (Figure 1). Once 1,25(OH)_2_D_3_ is formed in the kidneys it enters the circulation and travels to the small intestine to increase intestinal calcium absorption. If there is inadequate dietary calcium to satisfy the body’s requirement, 1,25(OH)_2_D_3_ can also interact with osteoblasts, inducing the production of receptor activator of NF-κB (nuclear factor kappa-light-chain-enhancer of activated B cells), which in turn initiates the amalgamation and formation of bone-resorbing osteoclasts [35] (Figure 1).

The genomic effects of 1,25(OH)_2_D_3_ begin with its binding to the vitamin D receptor (VDR), initiating a cascade of complex molecular interactions. These interactions result in the formation of a heterodimeric complex with the retinoid X receptor (RXR), an essential partner in mediating vitamin D signaling. Once bound, the 1,25(OH)_2_D_3_-VDR complex interacts with RXR to form the VDR-RXR heterodimer, which then recognizes and binds to specific DNA sequences known as vitamin D response elements (VDREs) located in the regulatory regions of target genes. When the VDR-RXR complex is anchored to the VDREs, it acts as a transcription factor that modulates the transcriptional activity of RNA polymerase II. This regulatory mechanism orchestrates the expression of a multitude of genes that are responsive to 1,25(OH)_2_D_3_, thereby influencing critical biological functions including calcium metabolism, bone health, immune system regulation, cell proliferation, differentiation, and angiogenesis [36,70,71,72,73,74].

1,25(OH)_2_D_3_ can also carry out biologic functions through a non-genomic pathway. 1,25(OH)_2_D_3_ binds to membrane-associated VDR, commonly referred to as the 1,25D-membrane-associated rapid response steroid-binding protein (1,25D-MARRS). This interaction occurs at the cell membrane, outside the nucleus, and triggers a cascade of rapid signaling events through direct protein–protein interactions among various intracellular molecules [75]. The activation of the non-genomic pathway facilitates the engagement of key signaling cascades involving molecules such as mitogen-activated protein kinases (MAPKs), phospholipase C (PLC),Ca^2+^-calmodulin-dependent protein kinase II (CaMKII), protein kinase C (PKC), protein kinase A (PKA), phosphatidylinositol-3 kinase (PI3K), and the Src family of protein tyrosine kinases. These kinases play a pivotal role in relaying signals to specific transcription factors, including RXR, SP1, and SP3 [36,75,76]. Once activated, these transcription factors also bind to VDREs located on the promoters of vitamin D-responsive genes, thereby modulating gene expression. The non-genomic pathway is characterized by its rapid response to 1,25(OH)_2_D_3_, facilitated through multiple protein–protein interactions. Additionally, vitamin D promotes the release of second messengers, including Ca^2+^, cyclic AMP, 3-phosphoinositides, and fatty acid. This cascade of signaling events contributes to a broader spectrum of cellular responses and physiological effects mediated by vitamin D, extending beyond the direct genomic actions traditionally associated with this vital hormone.

## 3. Mechanisms Associated with UVA/UVB-Induced Skin Cancers and Vitamin D’s Modulatory Effects

UVA and UVB irradiation drives carcinogenesis through a dual mechanism; it induces DNA damage that leads to mutations while impairing the host immune system’s ability to recognize and eliminate malignant cells. This process triggers oxidative stress, activates inflammatory pathways, and suppresses anti-tumor immune responses [77,78], which are not only implicated in skin cancer but also contribute to photoaging, a phenomenon characterized by premature skin aging, due to chronic UV exposure. Additionally, the probability of developing NMSC tends to increase with age, and skin type plays a crucial role. Individuals with lighter skin types are at higher risk due to reduced melanin concentrations, which offer less natural protection against UV irradiation.

At the molecular level, UVB radiation causes a range of DNA lesions, primarily by directly absorbing UVB photons by pyrimidine bases in the DNA. This results in the formation of various photoproducts, including pyrimidine dimers. UVB-induced DNA damage most commonly results in the formation of photoproducts between two adjacent pyrimidine bases on the same DNA strand, such as cytosine–cytosine (CC), thymine–thymine (TT), thymidine–cytosine (TC), and cytosine–thymidine (CT). These photoproducts are mainly cyclobutane pyrimidine dimers (CPDs) and 6-pyrimidine-4-pyrimidinone photoproducts (6–4 PPs) [49,79,80,81,82,83]. UVB radiation can also induce protein–DNA crosslinks and single-strand breaks, though these are less common than pyrimidine dimers.

Both CPDs and 6–4 PPs cause significant structural distortions in the DNA double helix, which impairs DNA replication and transcription, disrupting normal cellular function. The accumulation of these lesions results in an increased frequency of mutations, making them essential factors in UV-induced skin cancer [84]. Of the two lesions, 6–4 PPs are repaired more efficiently by cellular mechanism, while CPDs, particularly the TC and CC dimers, are more mutagenic.

The TP53 gene encodes the tumor suppressor and transcription factor p53, a key regulator of vital cellular processes, including cell cycle arrest, apoptosis, DNA repair, and cellular differentiation [85]. p53 is one of the most critical proteins in cancer biology, with a central role in controlling cancer initiation, progression, and dissemination. It is also the most frequently dysregulated protein in cancers, often through mutations [86]. In the context of skin cancer, mutations with a UVR signature in the TP53 gene are commonly observed and are essential contributors to skin tumorigenesis, particularly in NMSCs [86]. These mutations are often detected very early in pre-malignant lesions, indicating that they are major drivers of skin cancer development and progression [86,87]. UV-induced skin cancers often present C→T and CC→TT mutations in the p53 gene, commonly referred to as UV signature mutations [80,81,82,83,84,85]. p53 mutations play a significant role, as they are frequently associated with the development of skin cancers, especially SCC. Additionally, UV-induced mutations have also been found in other key tumor suppressor genes, such as PTCH1 (Patched1, a tumor suppressor gene; linked to BCC) and p16Ink4A (associated with melanoma), highlighting UV radiation as a key risk factor in the epidemiology of skin cancer [88]. If DNA lesions caused by UVB radiation are not effectively repaired, it can trigger cell death or provoke inflammatory responses in surrounding tissues, which contribute to further damage.

Moreover, errors in the DNA repair process may lead to the accumulation of mutations over time, thereby increasing the likelihood of skin cancer and other UV-related pathologies [89]. UVB radiation can also induce oxidative damage by causing guanine oxidation, leading to the formation of the purine photoproduct 8-hydroxy-2′-deoxyguanosine (8-OHdG). 8-OHdG serves as a biomarker of oxidative stress, indicating cellular damage resulting from reactive oxygen species. While it represents only a small portion of UVB-induced DNA damage, 8-OHdG can lead to G→T transversions, resulting in gene mutations that play a role in the carcinogenic process [90].

Beyond the aforementioned DNA lesions—CPDs, 6–4 PPs, and oxidative modifications—UVB radiation can induce other forms of DNA damage. These include protein–DNA cross-links, where proteins are covalently attached to DNA, disrupting essential cellular processes, as well as single-strand breaks, which compromise genomic integrity and contribute to the overall mutagenic burden [79]. Moreover, direct exposure to UV radiation can cause sunburn, photoaging, photoimmunosuppression, and inflammation, and induce genetic mutations that may lead to skin cancer [91].

UVA and UVB radiation penetrate through the ozone layer to the Earth’s surface at sufficient levels to impact human skin, contributing to photoaging [92]. UVA, being lower in energy but twenty times more prevalent in the atmosphere, is not blocked by glass and penetrates deep into the dermal layers, whereas UVB is mostly absorbed by the epidermis. UVA primarily causes indirect DNA damage and degrades collagen and elastin fibers through oxidative stress pathways, making it a key driver of photoaging [93]. Both UVA and UVB radiation contribute to DNA damage, oxidative stress, inflammation, and the breakdown of extracellular matrix (ECM) proteins, which collectively lead to the aged skin phenotype. However, UVB radiation has a unique role in synthesizing vitamin D_3_ in the skin. Vitamin D_3_ protects against photodamage by repairing CPDs, mitigating oxidative stress, and reducing chronic inflammation [94]. In contrast, UVA exposure, while contributing to skin damage, is incapable of producing the skin protective vitamin D_3_.

The intensity and biological impact of UVA and UVB radiation are influenced by several geographical factors, including latitude, altitude, air pollution, and seasonal variation, which contribute to regional differences in skin cancer prevalence. The amounts of UVA and UVB radiation fluctuate significantly based on latitude, time of day, season, and altitude [95]. UVA is always present when the sun is shining, from early morning when the sun rises until the sun sets, throughout the year, and therefore is less influenced by season and time of day compared to UVB radiation, which is significantly influenced by the zenith angle of the sun due to the efficiency of ozone absorbing it before it reaches the Earth’s surface. Therefore, although it would seem counterintuitive to recommend sensible sun exposure in the late morning until early afternoon instead of early morning and late afternoon during seasons when sufficient UVB radiation reaches the Earth’s surface, it is only in the late morning and early afternoon that cutaneous vitamin D_3_ synthesis occurs. In the early morning and late afternoon, the major UV component is UVA with little or no UVB. As a result, early morning and late afternoon sun exposure results in DNA damage and increased production of ROS with no benefit for producing vitamin D_3_ which can help mitigate DNA damage and ROS activity from exposure to UV radiation. The intensity of the solar UVA and UVB radiation that reaches the Earth’s surface peaks at midday and on the summer solstice [95]. People living closer to the equator or at higher altitudes are often at greatest risk of melanoma due to studies linking these geographical factors with increased UV exposure and melanoma rates worldwide [96]. Research has shown that melanoma risk is associated with average annual UV exposure and residential history, through the link between time spent outdoors and melanoma risk was significant only for individuals exposed at a young age [97]. Interestingly, Wong et al. [98] reported a higher incidence of melanoma among children in regions with low UV exposure compared to those in high UV exposure areas, although the reasons behind this observation remain unclear. It is suggested that sunburns in children from low UV exposure areas may result from intense, intermittent sun exposure, such as during vacations. This reasoning of intermittent sunburn experiences during childhood and early adulthood increasing risk for melanoma was further supported by the observation that occupational sun exposure reduces risk of melanoma [45].

## 4. Sunlight Dilemma: The Associations Between Sun Exposure, DNA Damage, and Skin Cancer Risk

Ranked as the fifth most common cancer globally, skin cancer has been recognized as one of the most impactful malignancies of the current decade [44,45,46,47,48,49,50,51,52,99]. It arises from various cell origins and is categorized into non-melanoma skin cancer and melanoma skin cancer. Non-melanoma skin cancer (NMSC), the most common form worldwide, originates in the epidermis and is categorized into basal cell carcinoma (BCC) and squamous cell carcinoma (SCC). BCC develops from basal cells in the epidermis, while SCC stems from keratinocytes within the same layer. In contrast, melanoma, the most aggressive form of skin cancer, originates from melanocytes situated at the junction of the epidermis and dermis [100,101,102].

NMSCs typically occur in sun-exposed areas of the body and are closely linked to chronic excessive sun exposure. NMSCs represent a significant health concern, being among the most common malignancies globally [103,104,105,106]. Between 2007 and 2017, the incidence of NMSCs increased by 33%, with cases reaching 7.7 million worldwide [105]. In Europe, the annual incidence of BCC has increased by 5% over the past decade [107], and studies suggest SCC incidence is rising and may soon rival that of BCC [108]. Data from Scotland, Germany, and Denmark report annual increases in BCC and SCC in the ranges 1.4–3.5%, 3.1–4.6%, and 3.3–11.6%, respectively, with Germany projecting a doubling of NMSC cases in the next decade [109]. In the United States, an estimated 3.6 million BCC cases and 1.8 million SCC cases are diagnosed annually [110]. The development of melanoma is strongly associated with childhood sun exposure resulting in sunburns, intermittent excessive sun exposure in young adults resulting in sunburning, being redheaded, having increased numbers of nevi, and genetic predisposition [45,111]. Melanoma, while comprising only approximately 4% of all skin cancers, is the leading cause of skin cancer-related deaths [77].

Chronic UV solar radiation exposure is the major causative factor in the development of NMSCs [112]. Unlike the link between NMSCs and chronic sun exposure, there remains controversy as to this link with melanoma. Interestingly, studies indicate that occupational sun exposure may reduce the risk of melanoma [1,45,113]. This suggests that the pattern and context of sun exposure play crucial roles in melanoma risk, highlighting the complex challenge of balancing adequate sun exposure for health benefits against the need to minimize risks associated with UV-induced skin pathologies.

Lindqvist et al. [114] examined the link between solar irradiation and all-cause mortality in the Melanoma in Southern Sweden (MISS) cohort, which included 29,518 Swedish women over a 20-year follow-up period. They found that avoiding sun exposure was linked to a reduced life expectancy of 0.6 to 2.1 years compared to those with the highest sun exposure. Remarkably, non-smokers who refrained from sun exposure had a life expectancy comparable to that of smokers with the highest levels of sun exposure, highlighting that avoiding sun exposure may pose a risk similar to smoking. The study suggested that the higher number of cancer deaths in the high sun exposure group was due to their longer overall life expectancy, rather than an increased risk of cancer itself. Overall, it underscores that vitamin D deficiency and sun avoidance are major health risk factors, comparable to smoking in their impact.

### 4.1. 1,25-Dihydroxyvitamin D_3_ and Its Inhibitory Effects on UVB-Induced Skin Carcinogenesis

Not only is the epidermis capable of synthesizing vitamin D_3_ upon exposure to solar UVB radiation, but it also possesses the enzymatic ability to convert vitamin D_3_ into its active form, 1,25(OH)_2_D_3_ [115]. Since the epidermis is a bloodless tissue, the likely major source of 1,25(OH)_2_D_3_ in the keratinocytes is from its local production [116]. Much debate has surrounded the necessity of sunlight exposure for humans, with the argument that the primarily benefit of sunlight is the production of vitamin D_3_ and that this can be obtained from the diet. However, we are beginning to appreciate that, as a result of solar UVB exposure, the skin has not only the capacity to produce the anti-proliferative hormone, 1,25(OH)_2_D_3_ but also several photoproducts of previtamin D_3_, lumisterol, and vitamin D_3_ which have biologic properties that help reduce the risk of developing NMSCs and melanoma [61,62,63].

1,25(OH)_2_D_3_ is a well-known pro-differentiating and anti-proliferative agent for keratinocytes and a variety of cancer cell lines. These biologic actions were translated into the development of 1,25(OH)_2_D_3_ and its active analogs for the treatment of the hyperproliferative skin disease psoriasis [117,118]. Vitamin D analogs such as calcipotriol, maxacalcitol, and tacalcitol are widely recognized for their therapeutic efficacy and safety in dermatological applications, particularly in the management of psoriasis. These compounds exhibit potent effects on keratinocyte proliferation and differentiation [119]. Calcipotriol (50 μg/g ointment), applied twice daily, has demonstrated slightly greater efficacy than betamethasone 17-valerate in treating psoriasis, with mild facial dermatitis reported in approximately 10% of cases [120]. Maxacalcitol (25 μg/g) has shown superior efficacy compared to once-daily calcipotriol, though burning sensations in treated lesions necessitated discontinuation in some patients [121]. Tacalcitol (4–20 μg/g) is generally well-tolerated, with transient irritation observed during initial treatment, leading to discontinuation in 5.9% of patients [122,123,124]. Adverse reactions to these vitamin D analogs, including allergic contact dermatitis, are infrequent. Collectively, these analogs represent reliable topical options for treating challenging skin conditions, offering sustained effectiveness without tachyphylaxis. Their development underscores the potential of targeted vitamin D pathways in mitigating inflammatory skin diseases with minimal systemic side effect [119,120,121,122,123,124]. Notably, 1,25(OH)_2_D_3_ has proven effective in treating psoriasis, and unlike active vitamin D analogs, this natural hormone causes less facial irritation compared to its analogs, making it a favorable option in certain dermatological treatments [125].

It has also been reported that the photoproducts 5,6-transvitamin D_3_ and tachysterol and their 25-hydroxy metabolites possess anti-proliferative effects in human keratinocytes similar to 1,25(OH)_2_D_3_ [126]. Additional studies have revealed that not only 1,25(OH)_2_D_3_ but also 1,25-dihydroxylumisterol_3_, a dihydroxy metabolite of the previtamin D photoproduct lumisterol_3_, are effective at inhibiting UV-induced damage in an immunocompetent mouse (Skh:hr1) model susceptible to UV-induced tumors [127]. Both 1,25(OH)_2_D_3_ and 1,25-dihydroxylumisterol_3_ significantly reduced UV-induced cyclobutane pyrimidine dimer (CPD), apoptotic sunburn cells, and immunosuppression. Furthermore, these compounds inhibited skin tumor development, both papillomas and squamous cell carcinomas, in mice. Dixon et al. [128] have suggested that lumisterol_3_ may be metabolized in keratinocytes to 1,25-dihydroxylumisterol_3_. They reported that 1,25-dihydroxylumisterol_3_ significantly reduced UV radiation-induced production of CPD, apoptotic sunburn cells, and immunosuppression similarly to 1,25(OH)_2_D_3_. Furthermore, they demonstrated that this compound inhibited skin tumor development, both papillomas and squamous cell carcinomas, in the immunocompetent mouse model SKH-hr1.

These photoproducts may also have the potential for reducing risk of deadly malignancies of the colon, prostate, and breast among other cells and organs, improving immune function to reduce autoimmune diseases and infectious diseases. A good example is in the multiple sclerosis mouse model where exposure to UVB radiation was more effective in reducing the development of multiple sclerosis-like disease when compared to giving this mouse model vitamin D_3_ [129]. Results revealing pro-differentiating and anti-proliferative properties of some of these photoproducts in skin cells, such as 1,25(OH)_2_D_3_, a well-known anti-proliferative agent, along with the demonstration that 1,25-dihydroxylumisterol_3_ has significant anti-photodamage and anti-carcinogenic effects in mouse models, strongly suggest that several of these photoproducts and/or their metabolites may have significant biologic and/or therapeutic effects.

**Figure 2:**
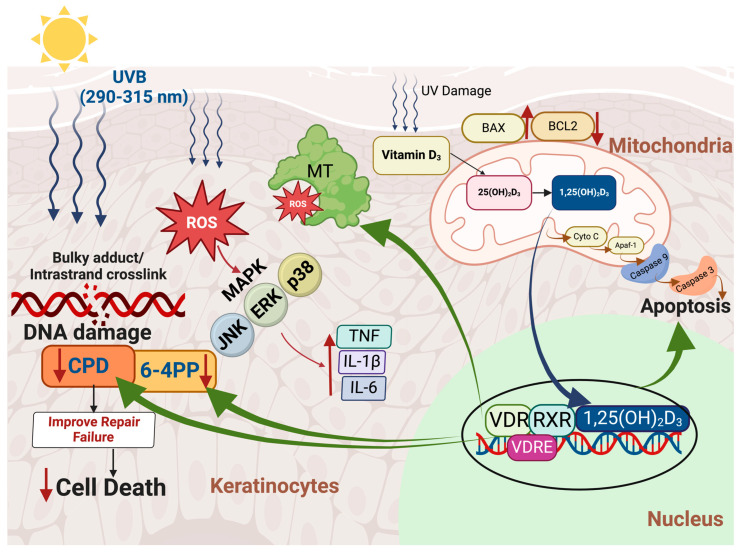
Multifaceted role of 1,25(OH)_2_D_3_ in mitigating oxidative stress, UVB-induced DNA damage, and apoptosis in keratinocytes. Keratinocytes not only produce vitamin D_3_ in response to ultraviolet B (UVB; 290–315 nm) radiation but also possess the enzymatic machinery to convert it into its active form, 1,25-dihydroxyvitamin D_3_ (1,25(OH)_2_D_3_). Solar UVB radiation induces reactive oxygen species (ROS) generation within epidermal kerotinocytes, reulting in oxidative stress and DNA damage, including bulky adducts and intrastrand crosslinks such as cyclobutane pyrimidine dimers (CPD) and 6-4 photoproducts (6-4PP). ROS activate mitogen-activated protein kinase (MAPK) signaling cascades including c-Jun N-terminal kinase (JNK), extracellular signal-regulated kinase (ERK), and p38 pathways, which amplify the expression of pro-inflammatory cytokines such as tumor necrosis factor (TNF), interleukin-1 beta (IL-1β), and interleukin-6 (IL-6). In mitochondria, UVB-induced oxidative stress disrupts the balance between pro-apoptotic (BAX) and anti-apoptotic (BCL2) proteins, facilitating the release of cytochrome c (Cyto C). This triggers apoptotic protease activating factor-1 (Apaf-1) activation and caspase-dependent apoptosis via caspase-9 and caspase-3. Metallothionein (MT), a ROS-scavenging protein, is upregulated as a defense mechanism to mitigate oxidative damage. The active form of vitamin D, 1,25(OH)_2_D_3_, enhances MT expression through its interaction with the vitamin D receptor (VDR) and retinoid X receptor (RXR). This complex modulates transcription by binding to vitamin D response elements (VDRE) in target genes. 1,25(OH)_2_D_3_ plays a protective role by reducing oxidative stress, limiting inflammatory responses, and sustaining MT function. 1,25(OH)_2_D_3_ significantly reduces the formation of CPDs and 6-4PP in keratinocytes following UVB exposure. Furthermore, 1,25(OH)_2_D_3_ promotes apoptosis in damaged cells, thereby preventing their transformation into malignant tumors. Green arrow represents the influence of 1,25(OH)_2_D_3_ on DNA damage, ROS and apoptosis. The up arrows (↑) and down arrows (↓) represent increases and decreases various biologic functions. Abbreviations: ROS, reactive oxygen species; 1,25(OH)_2_D_3_, 1,25-dihydroxyvitamin D_3_; CPD, cyclobutane pyrimidine dimers; 6-4PP, 6-4 photoproducts; MAPK, mitogen-activated protein kinase; JNK, c-Jun N-terminal kinase; ERK, extracellular signal-regulated kinase; TNF, tumor necrosis factor; IL-1β, interleukin-1 beta; IL-6, interleukin-6; BAX, pro-apoptotic protein; BCL2, anti-apoptotic protein; Cyto C, cytochrome c; Apaf-1, apoptotic protease activating factor-1; MT, metallothionein; VDR, vitamin D receptor; RXR, retinoid X receptor; VDRE, vitamin D response elements. Copyright Holick 2025.

1,25(OH)_2_D_3_ has demonstrated significant protective effects against UV radiation-induced cellular damage through its capacity to upregulate metallothionein [130,131], a protein with radical-scavenging properties that provides photoprotection [132]. This upregulation likely plays a significant role in reducing UVB-induced oxidative stress in cells (Figure 2).

**Figure 3:**
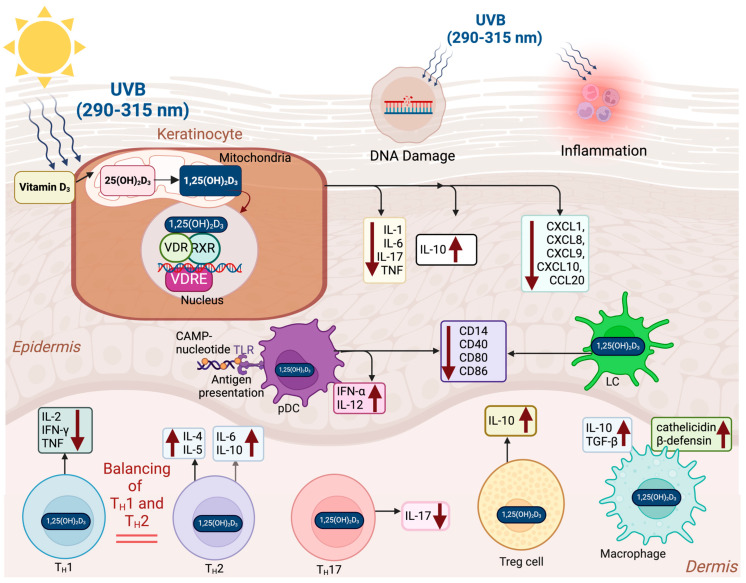
Interplay between UVB-induced vitamin D_3_ synthesis, keratinocyte signaling, and immunomodulatory effects of vitamin D. Solar ultraviolet B (UVB, 290–315 nm) radiation penetrates the epidermis, initiating vitamin D_3_ synthesis, DNA damage, and inflammation within the skin. Epidermal keratinocytes possess the enzymatic machinery to convert vitamin D_3_ into its active form, 1,25-dihydroxyvitamin D_3_ (1,25(OH)_2_D_3_). In the nucleus, 1,25(OH)_2_D_3_ binds to the vitamin D receptor (VDR), which forms a heterodimer with the retinoid X receptor (RXR). This complex modulates gene transcription by binding to vitamin D response elements (VDRE) in target genes. 1,25(OH)_2_D_3_ plays a pivotal role in maintaining immune homeostasis by modulating the activity of various immune cells that express the VDR. It suppresses the production of pro-inflammatory cytokines while promoting anti-inflammatory cytokine release, contributing to immune regulation and tumor suppression. In keratinocytes, 1,25(OH)_2_D_3_ influences the production of cytokines such as interleukin-1 (IL-1), interleukin-6 (IL-6), interleukin-10 (IL-10), interleukin-17 (IL-17), and tumor necrosis factor (TNF), as well as chemokines like chemokine (C-X-C motif) ligand 1(CXCL1), chemokine (C-X-C motif) ligand 8 (CXCL8), chemokine (C-X-C motif) ligand 9 (CXCL9), chemokine (C-X-C motif) ligand 10 (CXCL10), and chemokine (C-C motif) ligand 20 (CCL20). Notably, it decreases the expression of pro-inflammatory molecules (IL-1, IL-6, IL-17, TNF, CXCL1, CXCL8, CXCL9, CXCL10, and CCL20) while upregulating the anti-inflammatory cytokine IL-10, enhancing skin immune homeostasis. Dendritic cells (DCs), including Langerhans cells (LCs) and plasmacytoid dendritic cells (pDCs), are key antigen-presenting cells (APCs) in the skin. 1,25(OH)_2_D_3_ reduces the expression of activation markers such as CD14, CD40, CD80, and CD86 in DCs, promoting a tolerogenic state that mitigates excessive immune activation and autoimmunity. In pDCs, 1,25(OH)_2_D_3_ enhances the production of interferon alpha (IFN-α) and interleukin-12 (IL-12), crucial for anti-viral immunity. 1,25(OH)_2_D_3_ also balances the activities of T helper 1 cells (Th1) and T helper 2 cells (Th2) cells. Th1 cells produce interleukin-2 (IL-2), interferon gamma (IFN-γ), and TNF, driving pro-inflammatory responses against intracellular pathogens. In contrast, Th2 cells secrete interleukin-4 (IL-4), interleukin-5 (IL-5), IL-6, and IL-10, which support anti-inflammatory and allergic responses. By modulating Th1/Th2 balance, 1,25(OH)_2_D_3_ prevents chronic inflammation and immune suppression, maintaining immune equilibrium. Vitamin D suppresses T helper 17 (Th17) cell differentiation, reducing IL-17 production (a pro-inflammatory cytokine), and promotes Treg activity. Tregs secrete IL-10, fostering immune tolerance and reducing inflammation. This shift from Th17 to Treg dominance supports immune regulation. In macrophages, 1,25(OH)_2_D_3_ induces M2 polarization, increasing the production of anti-inflammatory cytokines such as IL-10 and TGF-β, which facilitate tissue repair and wound healing. Additionally, it stimulates the production of anti-microbial peptides (AMPs), including cathelicidin and β-defensin, by macrophages, bolstering the skin’s defense against infections. M2 macrophages also contribute to collagen synthesis and extracellular matrix formation, essential for tissue regeneration. The up arrows (↑) and down arrows (↓) represent increases and decreases in production and release of cytokines and other biologic agents by various cells associated with the skin in response to 1,25(OH)_2_D_3_ respectively. Abbreviations: UVB, ultraviolet B radiation; 1,25(OH)_2_D_3_, 1,25-dihydroxyvitamin D_3_; VDR, vitamin D receptor; RXR, retinoid X receptor; VDRE, vitamin D response elements; IL, interleukin; TNF, tumor necrosis factor; CCL, C-C motif chemokine ligand; CXCL, C-X-C motif chemokine ligand; DCs, dendritic cells; pDCs, plasmacytoid dendritic cells; IFN-α, interferon-alpha; TGF-β, transforming growth factor-beta; AMPs, anti-microbial peptides; Tregs, regulatory T cells; LCs, Langerhans cells; IL-12, interleukin-12; Th1, T helper 1 cells; Th2, T helper 2 cells; IFN-γ, interferon-gamma; Th17, T helper 17 cells. Copyright Holick 2025.

UVB radiation has been observed to suppress VDR gene expression [133], which is crucial for mediating the biological effects of 1,25(OH)_2_D_3_. Interestingly, 1,25(OH)_2_D_3_ and its analogues inhibit UVB-induced apoptosis. The likely reason is that they repair DNA damage, improving cellular health thereby reducing the need for apoptosis. 1,25(OH)_2_D_3_ and some active analogs also inhibit the pro-inflammatory interleukin-6 (IL-6) production in keratinocytes [134,135,136]. This complex interaction emphasizes the role of 1,25(OH)_2_D_3_ in reducing cellular damage and inflammation in response to UV exposure (Figure 2 and Figure 3).

Additionally, 1,25(OH)_2_D_3_ improves cellular health not only in human keratinocytes but also melanocytes and fibroblasts exposed to UV radiation. Wong et al. demonstrated that pre-treatment with 1,25(OH)_2_D_3_ and its active analogs significantly reduced CPD damage in skin cells exposed to UV radiation, achieving a decrease of up to 60% in a dose- dependent manner (10^−12^ to 10^−8^ M) [137]. Haes et al. reported that 1,25(OH)_2_D_3_ and its active analogs also significantly reduce the formation of CPDs in keratinocytes following UVB exposure [138]. Beyond its protective roles, 1,25(OH)_2_D_3_ has also been investigated for its effects on cancer cell behavior. In vitro studies and preclinical animal models have demonstrated that 1,25(OH)_2_D_3_ can influence cancer cell behavior by regulating differentiation, proliferation, and apoptosis, positioning it as a potential agent for cancer modulation [139]. Hager et al. showed that 1,25(OH)_2_D_3_ suppresses the growth of squamous cell carcinoma (SCC) cell lines by inducing p21- and p27-regulated cell cycle arrest in the G_0_/G_1_ phase, thus halting cell proliferation [140]. Similarly, Trémezaygues et al. demonstrated that 1,25(OH)_2_D_3_ at a concentration of 10^−7^ M inhibits proliferation and reduces the viability of cutaneous SCC cells [141]. These studies suggest that the active form of vitamin D may slow the progression of certain types of cancer by encouraging damaged cells to undergo apoptosis, thereby preventing them from developing into malignant tumors (Figure 3).

These findings collectively demonstrate that vitamin D_3_ produced in the skin and metabolized to 1,25(OH)_2_D_3_ plays a multifaceted role in counteracting oxidative stress, reducing UVB-induced DNA damage, and modulating inflammatory responses and apoptosis, while also having the potential to influence cancer progression. These properties underscore its promising role in preventing UV-induced skin carcinogenesis and regulating cancer cell behavior (Figure 2).

The VDR, a nuclear hormone receptor, functions as a transcription factor that mediates the genomic effects of vitamin D. These activities include regulating signaling pathways involved in apoptosis, cell cycle progression, and differentiation—key processes in the development and progression of various cancers [142]. Beyond the synthesis and activation of 1,25(OH)_2_D_3_, genetic variations in VDR, such as polymorphisms Bsm1, Apa1, Fok1, and Taq1, have been linked to modulation of skin health and susceptibility to skin cancer [143]. In a meta-analysis of ten studies comprising 5334 cases and 5466 controls, Fok1, Taq1, and Apa1 polymorphisms were identified as potential susceptibility biomarkers for skin cancer in Caucasian populations [144]. Another meta-analysis and systematic review [145] indicated that the VDR variants FokI, ApaI, and Bsml may influence the predisposition to developing cutaneous malignant melanoma.

### 4.2. Vitamin D and the Anti-P53 Connection

The dual nature of UVB, stimulating vitamin D_3_ synthesis while at the same time increasing risk of carcinogenesis, is further explored by Kanno et al. [146] and Holick [147]. Kanno et al. conducted a post hoc analysis of the AMATERASU randomized clinical trial, which investigated the impact of 2000 IU daily vitamin D_3_ supplementation in patients with digestive tract cancer exhibiting p53-immunoreactivity [146]. This subgroup was identified by the presence of anti-p53 antibodies in serum and the nuclear accumulation of p53 in over 99% of cancer cells, indicating p53 missense mutations. In patients with detectable anti-p53 antibodies, vitamin D supplementation significantly improved relapse-free survival, reducing the risk of relapse or death by 27% compared to the placebo group. However, no significant improvement in 5-year relapse-free survival was observed in patients without p53 immunoreactivity, indicating that the benefits of vitamin D supplementation may be specifically associated with p53-mutated cancers.

This observation [146] provides valuable insight into the relationship between vitamin D status and cancer outcomes, suggesting that vitamin D supplementation may be beneficial for patients with p53 mutations, enhancing cancer remission and reducing mortality. Mutations in p53 are present in approximately 50% of human cancers, resulting in the accumulation of mutant p53 (mutp53). This mutant form not only loses its tumor-suppressive capabilities but also interferes with the function of wild-type p53. Remarkably, mutp53 has been found to bind to the promoter region of VDR-responsive elements, potentially creating an anti-apoptotic environment. This binding may reduce the ability of 1,25(OH)_2_D_3_-VDR to promote the expression of pro-apoptotic genes, thereby diminishing vitamin D’s protective efficacy [148,149].

It is clear that vitamin D supplementation in patients with p53-immunoreactive tumors leads to a more than 2.5-fold improvement in relapse-free survival compared to a placebo. This highlights the importance of maintaining adequate vitamin D status to enhance the immune response, support normal cell differentiation, and improve outcomes in patients with certain genetic profiles, such as those involving p53 mutations [147]. However, in patients without p53 immunoreactivity, vitamin D supplementation has not shown significant benefits in terms of relapse-free survival, reinforcing the need for individualized treatment strategies based on genetic characteristics.

These observations illustrate the complex and context-dependent role of vitamin D in skin carcinogenesis. While 1,25(OH)_2_D_3_ shows protective effects through anti-inflammatory, anti-apoptotic, and anti-oxidative mechanisms, its efficacy appears to be modulated by the mutational status of p53. This observation may have relevance for NMSCs since a major cause of keratinocytes becoming malignant is due to the UVB-induced damage of both p53 genes in a keratinocyte. The mechanisms that trigger the human body to produce antibodies to mutant p53s or factors that may prevent their production remain poorly understood. It is well established that 1,25(OH)_2_D_3_ is an effective modulator of B lymphocyte function and therefore improvement in vitamin D status through sunlight exposure may help activate B lymphocytes to produce antibodies, like antibodies to mutant p53 proteins, to fight skin cancers including NMSCs, which are often initiated by mutations of the p53 gene [150].

Adequate vitamin D status plays a crucial role in reducing the risk and improving the prognosis of melanoma and non-melanoma skin cancers. Research indicates that sufficient vitamin D status is linked to a reduced risk of melanoma occurrence, with a relative risk of (RR) 0.62 [95%CI: 0.42–0.94], emphasizing its preventive potential [151]. Regarding melanoma prognosis, studies demonstrate that lower serum 25(OH)D_3_ concentrations correlate with unfavorable prognostic traits such as increased Breslow thickness and reduced melanoma survival, even after accounting for inflammatory biomarkers [152]. Genetic variations influencing vitamin D binding protein levels have also been linked to poorer melanoma-specific survival, further highlighting the importance of vitamin D status [153]. Moreover, decreased vitamin D status at diagnosis is significantly associated with adverse tumor characteristics, including higher mitotic rates, ulceration, and reduced overall survival [154,155]. A recent meta-analysis further supports the link between low vitamin D status and both increased melanoma risk and worsened prognosis, underscoring the protective role of vitamin D against skin cancer [156]. Collectively, these findings highlight the multifaceted effect of vitamin D on skin cancer progression and prevention.

### 4.3. Immunomodulatory Effects of Vitamin D

Cutaneous T cells are a heterogeneous group of immune cells present in the skin, including Th1, Th2, Th3, Th9, Th17, Th22, Th25, and regulatory T cells (Tregs) [157]. The VDRs in T cells interact with 1,25(OH)_2_D_3_, modulating the secretion of cytokines that decrease inflammatory activity and initiate immune tolerance [157,158]. Mast cells in the skin can be stimulated to release biologically active factors involved in the modulation of tumor growth [157,158,159,160,161]. Depending on the circumstances they can release pro- or anti-inflammatory mediators as well as pro-angiogenic and growth factors that could potentially stimulate cancer growth [161]. 1,25(OH)_2_D_3_ binds to the VDRs in mast cells that provide a braking mechanism that reduces the production of pro-inflammatory cytokines and promotes release of anti-inflammatory cytokines, thereby inhibiting tumor growth [161,162] (Figure 2 and Figure 3).

1,25(OH)_2_D_3_ plays an essential role in maintaining immune homeostasis by modulating the activity of various cells (Figure 3). Specifically, 1,25(OH)_2_D_3_ enhances CCR10 expression in T cells, promoting skin-specific T cell homing and effective immune surveillance in the skin [163,164]. Vitamin D suppresses Th17 cell differentiation, reducing the production of IL-17, a key pro-inflammatory cytokine, and promotes the activity of Tregs, which produce IL-10 to support immune tolerance. This shift from Th17 to Treg activity helps in reducing excessive inflammation and promoting immune regulation.

Dendritic cells (DCs), including Langerhans cells (LCs) and plasmacytoid dendritic cells (pDCs), are crucial antigen-presenting cells (APCs) in the skin that interact with 1,25(OH)_2_D_3_. 1,25(OH)_2_D_3_ reduces the expression of activation markers such as CD14, CD40, CD80, and CD86 in DCs, promoting a tolerogenic state that helps in preventing autoimmunity by reducing excessive immune activation [165,166]. In pDCs, a 1,25(OH)_2_D_3_ analog, calcipotriol, enhances the production of IFN-α and IL-12, which are important for anti-viral immunity [165]. Langerhans cells, as specialized APCs, are modulated by 1,25(OH)_2_D_3_ to adopt a tolerogenic role, further promoting immune balance in the skin. Dam et al. demonstrated that both 1,25(OH)_2_D_3_ and its analog, calcipotriol, decrease the number of CD1a+ DCs and alter their morphology, reducing antigen presentation and immune activation [165]. This supports the immunosuppressive role of vitamin D in the skin.

1,25(OH)_2_D_3_ balances the activities of Th1 and Th2 cells. Th1 cells produce IL-2, IFN-γ, and TNF, contributing to pro-inflammatory responses that combat intracellular pathogens. Th2 cells, on the other hand, release IL-4, IL-5, IL-6, and IL-10, which contribute to anti-inflammatory and allergic responses. By balancing Th1 and Th2 activities, vitamin D helps prevent chronic inflammation and immune suppression, maintaining overall immune equilibrium [159] (Figure 3).

1,25(OH)_2_D_3_ also significantly affects keratinocytes, which produce cytokines such as IL-1, IL-6, IL-10, IL-17, IL-18, IL-22, and TNF, as well as chemokines like CXCL1, CXCL8, CXCL9, CXCL10, and CCL20. These molecules play a crucial role in coordinating immune responses in the skin [7]. Vitamin D enhances the production of anti-microbial peptides (AMPs) such as cathelicidin and β-defensin by keratinocytes, strengthening the skin’s defense against infections [167] (Figure 3).

Moreover, vitamin D influences macrophages, favoring their polarization to the M2 phenotype, which secretes anti-inflammatory cytokines such as IL-10 and TGF-β, facilitating tissue repair and wound healing [168]. M2 macrophages also contribute to collagen synthesis and extracellular matrix formation, which are vital during tissue healing.

In summary, these immunomodulatory effects of 1,25(OH)_2_D_3_ highlight its potential benefits in skin cancer prevention and treatment. By balancing immune activation and tolerance, 1,25(OH)_2_D_3_ reduces excessive inflammation, which is crucial in preventing the inflammatory environment that can cause cancer initiation and progression (Figure 3).

## 5. Balancing Skin Protection and Vitamin D_3_ Synthesis: Strategies for Safe Sun Exposure

Exposure to sunlight is a well-established method for stimulating cutaneous production of vitamin D_3_, as UVB radiation catalyzes the conversion of 7-DHC to previtamin D_3_ in the skin. Determining the ideal amount of UV exposure needed to maintain adequate serum vitamin D concentrations while simultaneously minimizing the risk of associated skin cancer remains a significant challenge. A crucial factor in maintaining tissue homeostasis, vitamin D operates through several mechanisms, including promoting tissue repair, downregulating pro-inflammatory mediators, and inhibiting the progression and proliferation of various cancers [1,169]. These multifaceted effects underscore the importance of balancing UV exposure for health benefits while mitigating potential risks.

A variety of factors influence the synthesis of vitamin D through solar UVB exposure, making it challenging to establish a universal recommendation for optimal UV exposure. It is generally recommended to expose skin areas such as the arms, legs, abdomen, thorax, and back to a suberythemal dose of sunlight for effective vitamin D_3_ production. However, protection of the face with a hat and/or sunscreen is advisable due to chronic facial sun exposure increasing susceptibility to sun damage. The face only constitutes approximately 4% of the body surface, contributing minimally to vitamin D_3_ synthesis. Exposure of a healthy young adult in a bathing suit to simulated sunlight equivalent to 1 minimal erythemal dose (MED) has been shown to raise circulating 25(OH) D_3_ concentrations, comparable to the intake of 15,000–20,000 IU of vitamin D_3_. Thus, exposure of approximately 20% of the body surface to 0.5 MED is estimated to produce 1500–2000 IU of vitamin D in a healthy young adult. This amount is considered sufficient according to the Endocrine Society’s 2011 vitamin D guidelines [70]. For individuals at higher risk of skin cancer or those living in regions with limited sunlight, alternative strategies, including vitamin D supplementation, can maintain optimal serum 25(OH)D_3_ concentrations without relying solely on UV exposure.

Emerging evidence also highlights the role of dietary sources of vitamin D, such as fortified foods, oily fish, cod liver oil, and sun-dried mushrooms, as effective adjuncts to UV exposure in achieving adequate circulating concentrations of 25(OH)D_3_. Additionally, wearing protective clothing and using physical barriers, like hats or umbrellas, can reduce the cumulative UV burden on the skin while allowing controlled exposure to sunlight for vitamin D_3_ synthesis. These strategies collectively emphasize the feasibility of maintaining sufficient vitamin D status while adhering to skin cancer prevention measures [1]. In addition, the app dminder.info (ontometrics.com, accessed on 10 January 2025), available for iPhone and iPad, can help users track their sun exposure and optimize vitamin D_3_ synthesis based on personalized factors such as skin type and location, further emphasizing the feasibility of maintaining sufficient vitamin D status while adhering to skin cancer prevention measures. The app also alerts the user to overexposure to solar radiation, thereby decreasing the risk of sunburn. Another strategy is to minimize early morning and late afternoon sun exposure that only provides UVA radiation that increases the risk of skin cancer and skin damage while providing no benefit for producing vitamin D_3_ that helps to counteract solar-induced production of ROS and DNA damage.

## 6. Conclusions

Vitamin D_3_, synthesized in the skin through solar UVB exposure, plays a pivotal role in maintaining health and potentially mitigating non-melanoma and melanoma cancer risk [155]. While excessive sun exposure is linked to skin cancer through direct DNA damage and oxidative stress, insufficient UVB exposure limits the production of vitamin D_3_, contributing to broader public health concerns [34]. The dual role of exposure to solar UVB radiation in inducing skin carcinogenesis while at the same time promoting the production of vitamin D_3_ as a protector of skin health through anti-inflammatory activity and DNA repair mechanisms highlights the need for a balanced approach to recommendations for sensible sunlight exposure.

Globally, the understanding of this delicate balance is critical. The British Association of Dermatologists, Cancer Council Australia, and the World Health Organization recognize the importance of sensible sun exposure to optimize vitamin D_3_ synthesis while minimizing skin cancer risks [170,171,172]. Their recommendations underscore the necessity of individualized sunlight exposure guidelines that account for factors such as skin type, geographic location, and lifestyle.

By adopting this balanced strategy, public health efforts can better navigate the intricate relationship between sunlight, vitamin D_3_ synthesis, and skin cancer prevention, ensuring both health optimization and cancer risk reduction.
